# Interstitial Cells and Bladder Pathophysiology—Passive Bystanders or Active Participants?

**DOI:** 10.1016/j.juro.2011.02.2587

**Published:** 2011-05

**Authors:** Karen D. McCloskey

**Affiliations:** Centre for Cancer Research and Cell Biology, School of Medicine, Dentistry and Biomedical Sciences, Queen's University, Belfast, Northern Ireland, United Kingdom

Interstitial cells (ICs) in the bladder have been investigated for more than a decade by many independent groups throughout the world. These efforts have provided compelling evidence that the bladder wall contains subpopulations of ICs in the mucosal and detrusor regions that are structurally associated with urothelial cells, smooth muscle cells and nerves, with some populations comprising interconnected networks.^1^ ICs have been visualized in human and animal tissues using markers such as vimentin or KIT antibodies, and have been characterized ultrastructurally with transmission electron microscopy.

Physiological studies of IC subtypes in the bladder concur that these novel cells may contribute to the complex cellular signaling within the bladder wall that underpins normal bladder function. Davidson and McCloskey hypothesized that ICs may form a functional conduit for the transmission of signals from the urothelium to other cells within the bladder wall.^2^ Moreover others have suggested a role for ICs in the sensory response to stretch during filling.^3^ ICs have been broadly considered to act by pacemaking or modulating detrusor smooth muscle spontaneous activity within the normal bladder.^1^ In support of this idea mucosal and detrusor ICs exhibit spontaneous electrical and Ca^2+^ signaling profiles which may be commensurate with pacemaking. However, there is currently insufficient direct evidence that ICs modulate the activity of normal detrusor smooth muscle.

The role of ICs in the bladder may actually be more apparent in pathophysiological conditions such as obstructed bladder, neurogenic bladder or painful bladder syndrome/interstitial cystitis, all of which are associated with detrusor overactivity. Increased numbers of ICs have been shown in samples of overactive human bladder,^4^ and augmented connexin 43 labeling associated with mucosal ICs has been reported in human^5^ and rat neurogenic bladders.^6^ Kubota et al reported a marked increase in suburothelial and subserosal IC populations in guinea pig obstructed bladder.^7^ Their findings were consistent with the recent work of Grol et al in this issue of The Journal (page 1959), who further demonstrated M3 muscarinic receptor expression on bladder ICs. A study of mucosal (suburothelial) ICs in bladder samples from patients with neurogenic detrusor overactivity and painful bladder syndrome demonstrated ultrastructural and morphological changes resulting in transformation to a more fibroblast-like phenotype.^8^

Observations of enhanced IC numbers in bladders from patients or animal models with an overactive phenotype raise the question of whether these 2 phenomena may be linked. It is not yet clear whether defects in IC subpopulations contribute to the cause of bladder dysfunction or whether they occur as a consequence of bladder wall remodeling, eg hypertrophy, which typically accompanies neurogenic or obstructed bladder. In vitro tension recordings from overactive human bladder tissues with enhanced numbers of ICs showed increased contractility which was more sensitive to imatinib mesylate (Gleevec®, which targets KIT-positive ICs) than the activity of normal tissues.^4^ Gleevec was also reported to reduce spontaneous contractions of bladders from spinal cord injured rats in contrast to controls, where there was little effect.^9^ Interestingly the opposite situation has been found in the underactive bladder phenotype seen in patients with megacystis-microcolon intestinal hypoperistalsis syndrome in which the typical unobstructed, distended bladder lacked ICs compared with control tissues.^10^

The current literature consistently indicates that changes in the numbers of IC subpopulations are associated with dysfunctional phenotypes, giving support to the notion that ICs may be responsible for bladder pathophysiology and, therefore, may present novel therapeutic targets for the treatment of dysfunctional bladder. More research is needed in this area to address the caveats associated with considering ICs as potential treatment options. Appropriately powered studies on IC expression and activity in patient tissues matched with clinical urodynamic assessment and evaluation of reported symptoms are not yet available. There are also limitations in translating basic research findings from animal models to patient symptoms. The apparent correlation between bladder dysfunction and alterations in IC numbers is unlikely to fully account for aberrant bladder activity, which has other credible explanations unrelated to ICs such as smooth muscle hypertrophy, enhanced coupling between smooth muscle cells, connective tissue remodeling, changes in afferent signaling or altered release of factors from the urothelium.

In summary, we currently understand ICs as modulators of smooth muscle activity in the normal bladder by integrating and communicating information from urothelial cells, smooth muscle cells, nerves and microvessels. Their role may be relatively minor, acting as passive bystanders until stimulated by changes in the bladder microenvironment to modify the activity of neighboring cells in response. The function of these intriguing cells appears to be more dynamic in bladder pathophysiology where there is convincing evidence that changes in their numbers accompany an overactive or underactive phenotype. Ongoing and future work to investigate the physiological properties of ICs (eg ion channel activity, receptor signaling, Ca^2+^signaling) and their interactions with other cells in the diseased bladder wall has the potential to better explain the cellular basis of the dysfunctional bladder.
